# The psychological benefits of outdoor landscape sketching in higher art education: an extended SOR perspective

**DOI:** 10.3389/fpsyg.2026.1884307

**Published:** 2026-07-24

**Authors:** Mengqi Liu, Lei Feng

**Affiliations:** 1 Department of Environmental Design, College of Design and Art, Taiyuan University of Science and Technology, Taiyuan, Shanxi, China; 2 School of Foreign Languages, Soochow University, Suzhou, China; 3 Chongqing New Guidance Intelligent Technology Research Institute Co., Ltd, Chongqing, China

**Keywords:** art education, artistic creation, outdoor landscape sketching, perceived emotional healing, perceived personal growth, Stimulus-Organism-Response (S-O-R) theory

## Abstract

**Introduction:**

Outdoor landscape sketching is widely used in higher art education to develop students’ artistic skills and aesthetic perception. However, its potential psychological benefits and the mechanisms linking environmental experiences with students’ emotional and developmental outcomes remain insufficiently understood. This study extended the Stimulus–Organism–Response (SOR) framework by incorporating perceived emotional healing and perceived personal growth, proposing a dual-pathway model in which artistic creation mediates the relationships between environmental emotions and perceived psychological benefits.

**Methods:**

A cross-sectional survey was conducted among 350 undergraduate art and design students from three higher education institutions in China who had participated in outdoor landscape sketching courses. Data were collected using an adapted five-point Likert questionnaire measuring emotions in natural environments, emotions in cultural environments, artistic creation, perceived emotional healing, and perceived personal growth. Exploratory factor analysis and partial least squares structural equation modeling (PLS-SEM) were used to analyze the data.

**Results:**

Emotions in natural environments were positively associated with artistic creation (*β* = 0.351, *p* < 0.001) and perceived emotional healing (*β* = 0.197, *p* = 0.002). Emotions in cultural environments were positively associated with artistic creation (*β* = 0.495, *p* < 0.001) but were not directly associated with perceived emotional healing (*p* = 0.497). Artistic creation was positively associated with perceived emotional healing (*β* = 0.692, *p* < 0.001), which in turn was positively associated with perceived personal growth (*β* = 0.879, *p* < 0.001). In addition, artistic creation significantly mediated the relationships between environmental emotions and perceived emotional healing in both natural and cultural environments.

**Discussion:**

The findings suggest that artistic creation represents an important mechanism through which environmental experiences are associated with students’ perceived emotional healing and personal growth during outdoor landscape sketching courses. By extending the SOR framework with a dual-pathway mediation model, this study advances understanding of the psychological value of arts-based educational activities and provides practical implications for integrating experiential landscape sketching into higher art education to support students’ emotional well-being and personal development.

## 1 Introduction

In recent years, the mental health issues among adolescents and young adults worldwide have become a growing global concern. A recent study published in *JAMA Psychiatry* estimated that approximately 293 million individuals aged 5–24 globally suffer from at least one mental health disorder, with a prevalence rate of 13.63% among those aged 20–24 (Kieling et al., 2024). In China, problems such as anxiety, depression, and stress-related disorders are particularly prominent among university students (Chen et al., 2022). Consequently, promoting student well-being and identifying effective educational approaches to support students’ psychological health have become important priorities in higher education.

In response to this challenge, increasing attention has been directed toward innovative educational approaches that support students’ psychological well-being alongside academic development. Among these approaches, art education has attracted growing interest because of its potential to facilitate emotional well-being and self-expression (Hannigan et al., 2019). However, current research has largely focused on clinical art therapy, whereas the psychological value of art-related learning experiences in formal educational settings remains underexplored (Kroyanker et al., 2018). This gap highlights the need to investigate how educational art activities may contribute to students’ emotional well-being and personal development.

Outdoor Landscape Sketching is a foundational practice-oriented course commonly offered in undergraduate art and design education. This course guides students to immerse themselves in both natural and cultural environments, thereby developing their observational, representational, and aesthetic skills while simultaneously experiencing diverse emotional and sensory stimuli. Beyond its educational function, Outdoor Landscape Sketching may also provide psychological benefits through stress reduction, emotional regulation, and enhanced self-awareness (Stuckey and Nobel, 2010). Artistic activities in outdoor environments have further been associated with enhanced emotional management and personal reflection (Malchiodi, 2012). Therefore, the course provides a unique educational context in which environmental experiences, artistic creation, and perceived psychological outcomes may be closely interconnected (Malchiodi, 2012).

Despite these potential benefits, existing studies on outdoor landscape sketching have primarily focused on artistic skill development, aesthetic training, and learning outcomes, while the potential psychological significance of environmental experiences has received comparatively limited attention (Chen et al., 2022; Wang, 2022). Moreover, several important gaps remain. First, the distinct roles of natural and cultural environments in shaping students’ emotional and artistic experiences have not been systematically examined. Second, although artistic creation is considered a central component of landscape sketching, its role as a potential mediator linking environmental experiences with psychological outcomes remains underexplored. Specifically, there is limited empirical evidence regarding whether and how artistic creation mediates the associations between environmental experiences and perceived emotional healing.

To address these gaps, this study adopts the Stimulus-Organism-Response (SOR) framework to investigate the relationships among environmental experiences in natural and cultural environments (Stimulus), artistic creation (Organism), and perceived emotional healing (Response) within the outdoor landscape sketching course. Building upon the traditional SOR framework, the study further incorporates perceived personal growth as a Consequence outcome to examine the broader developmental implications of artistic engagement. Additionally, the study examines whether artistic creation serves as a mediating mechanism through which environmental experiences contribute to students’ perceived emotional healing and personal growth.

Accordingly, this study aims to address the following three research questions:

1. How do emotional experiences in natural and cultural environments relate to students’ artistic creation through landscape sketching courses?
2. How are artistic creation and environmental experiences during outdoor landscape sketching associated with students’ perceived emotional healing and personal growth?
3. Does artistic creation mediate the relationships between emotional experiences and perceived emotional healing?

## 2 Literature review

### 2.1 Natural environments, emotional experiences, and artistic creation

Natural environments provide rich sensory experiences through their visual beauty, sounds, spatial openness, and atmospheric qualities. According to Attention Restoration Theory (ART), exposure to natural environments reduces mental fatigue and restores depleted cognitive resources (Kaplan and Kaplan, 1989). Empirical studies have consistently shown that contact with nature promotes positive emotional outcomes and psychological well-being. For instance, natural environments have been shown to enhance positive emotions, particularly among urban populations, while also reducing anxiety and stress-related symptoms (Bratman et al., 2019; White et al., 2021). Moreover, awe-inspiring natural scenery, such as mountains, waterfalls, and starry skies, can evoke strong feelings of fascination, wonder, and emotional elevation (Pan et al., 2024).

These positive emotional experiences may play an important role in motivating artistic creation (Green and Rosenkranz, 2025). Feelings of awe, fascination, and emotional resonance can heighten individuals’ sensitivity to environmental details, encouraging deeper observation and aesthetic appreciation of the surrounding landscape (Harrison, 2023). Through this process, emotional experiences generated through encounters with natural environments may be transformed into artistic inspiration and creative expression.

Studies in art education further suggest that direct interaction with natural environments can enrich artistic practice. Exposure to nature has been found to enhance aesthetic sensitivity and facilitate more flexible and expressive use of color (Al-Husiny, 2024; Zhang and Yu, 2014). Wango (2022) further argued that direct observation of natural scenery provides diverse visual stimuli that strengthen artistic perception, thereby supporting creative expression. Taken together, natural environments function not only as sources of visual inspiration but also as emotional catalysts that facilitate artistic creation.

Therefore, the following hypothesis is proposed:

*H1*: The positive emotional experiences evoked by natural environments during outdoor landscape sketching courses positively influence students’ engagement in artistic creation.

### 2.2 Cultural environments, emotional experiences, and artistic creation

In addition to natural environments, cultural environments can generate emotionally meaningful experiences through their historical, social, and symbolic significance (Wu et al., 2025a). In this study, cultural environments encompass traditional villages, vernacular architecture, historic streets, museums, heritage sites, urban landmarks, and other places that embody collective memory and cultural identity. Unlike natural environments, whose influence is often derived from sensory and restorative experiences, cultural environments evoke emotions through the meanings, narratives, and values embedded within them (Bai et al., 2025).

Cultural environments are often appreciated not only for their physical appearance but also for the historical meanings, cultural traditions, and collective memories they embody. As Taylor and Lennon (2012) argued, aesthetic appreciation of cultural landscapes is shaped by the cultural meanings and symbolic values embedded within them rather than by visual characteristics alone. For example, in the Chinese context, traditional villages, vernacular architecture, historic streets, and heritage sites embody longstanding cultural traditions, regional identity, and historical continuity. Consequently, university students participating in outdoor landscape sketching may interpret these environments through culturally shared meanings, fostering stronger cultural resonance, place attachment, and aesthetic appreciation (Zhou and Mohamed Afla, 2026), which in turn may enhance their emotional engagement with the environment.

Previous studies have shown that culturally meaningful environments can elicit a range of positive emotional responses, including nostalgia, cultural resonance, place attachment, and identity affirmation. For example, museum environments have been found to promote psychological restoration through experiences of soft fascination (Packer and Bond, 2010). Similarly, heritage sites and traditional cultural landscapes can evoke feelings of nostalgia and strengthen cultural identity by connecting individuals with shared histories and collective memories (Sang, 2024; Tian et al., 2024). These emotionally rich experiences allow individuals to engage with cultural environments not merely as physical spaces but as meaningful representations of cultural values and historical narratives.

Such emotionally meaningful experiences may contribute to artistic creation by enriching individuals’ aesthetic appreciation and artistic creation. Emotions such as nostalgia, cultural resonance, and attachment to place encourage students to move beyond the mere depiction of physical scenery and engage with the symbolic meanings, historical narratives, and cultural values embedded within cultural environments (Segers et al., 2021). Through this process, cultural environments provide not only visual stimuli but also emotional and interpretive resources that can inspire creative thinking and deepen creative expression (Fadhlan, 2025). Accordingly, emotional experiences derived from cultural environments may serve as an important psychological foundation for artistic creation during landscape sketching activities.

Therefore, the following hypothesis is proposed:

*H2*: The positive emotional experiences evoked by cultural environments during outdoor landscape sketching courses positively influence artistic creation.

### 2.3 Emotional experiences and emotional healing

Positive emotional experiences play an important role in promoting psychological well-being and emotional recovery. According to the Broaden-and-Build Theory, positive emotions expand individuals’ cognitive and behavioral resources, helping them cope more effectively with stress and adversity while fostering psychological resilience (Fredrickson, 2001). Empirical research has further demonstrated that positive emotional states contribute to improved well-being and psychological resilience (Kwon and Lee, 2022). Conversely, persistent negative emotions and maladaptive processes such as rumination often intensify stress and impair psychological functioning (Watkins and Roberts, 2020).

Within outdoor landscape sketching courses, both natural and cultural environments may generate a variety of positive emotional responses, including awe, tranquility, nostalgia, cultural resonance, and a sense of belonging (Zhang et al., 2025). These emotional experiences may help students temporarily disengage from daily stressors, and facilitate psychological restoration, thereby contributing to emotional recovery (Pouya and Ergan, 2025). Previous studies have shown that emotionally meaningful experiences in both natural and cultural environments are associated with enhanced well-being and restorative outcomes (Xu et al., 2025; Wang et al., 2026; Wu et al., 2025b). Therefore, positive emotional experiences elicited during landscape sketching may serve as an important pathway through which environmental engagement contributes to emotional healing.

Accordingly, the following hypotheses are proposed:

*H3*: The positive emotional experiences evoked by natural environments during outdoor landscape sketching courses positively influence perceived emotional healing.

*H4*: The positive emotional experiences evoked by cultural environments during outdoor landscape sketching courses positively influence perceived emotional healing.

### 2.4 Artistic creation and emotional healing

Artistic creation has long been recognized as an effective pathway to emotional healing. For example, participation in artistic activities such as painting has been associated with enhanced mindfulness, reduced physiological stress, and improved emotional well-being (Kaimal et al., 2016). From a psychophysiological perspective, repetitive and focused creative activities activate the relaxation response and promote psychological calmness (Benson and Klipper, 2000). Chilton (2013) also suggested that artistic engagement frequently induces flow experiences characterized by enjoyment, concentration, and a sense of accomplishment, all of which contribute to emotional well-being.

Within landscape sketching courses, students actively transform their environmental perceptions and emotional experiences into visual expression. For instance, Wango (2022) suggested that participants engaged in on-site landscape painting naturally integrate their emotions, reflections, and environmental experiences into the artwork. Here, artistic creation also functions as a means of emotional processing and self-expression, facilitating emotional healing and psychological restoration. McNiff (2004) further emphasized that intuitive artistic expression allows individuals to release emotional tensions and initiate healing processes.

Furthermore, because environmental emotions are often interpreted, expressed, and reconstructed through artistic activities (Kim et al., 2026), artistic creation may serve as a key mechanism linking emotional experiences with emotional healing. Through this transformative process, students actively process their emotional responses, which in turn facilitates psychological recovery. By transforming emotions into creative expression, students may gain emotional release, self-understanding, and psychological recovery (Song and Jang, 2026). Therefore, artistic creation is expected to mediate the relationship between environmental emotions and emotional healing.

Accordingly, the following hypotheses are proposed:

*H5*: Artistic creation during outdoor landscape sketching courses positively influences perceived emotional healing.

*H5a*: Artistic creation mediates the relationship between students’ positive emotional experiences in natural environments and their perceived emotional healing during outdoor landscape sketching courses.

*H5b*: Artistic creation mediates the relationship between students’ positive emotional experiences in cultural environments and their perceived emotional healing during outdoor landscape sketching courses.

### 2.5 Emotional healing and personal growth

Emotional healing may extend beyond immediate psychological recovery and contribute to broader processes of personal growth and self-development. According to psychological well-being theory, personal growth reflects an individual’s openness to new experiences, continuous self-development, and realization of personal potential (Ryff, 1989). Emotional healing can create favorable psychological conditions for such development by reducing emotional distress and restoring individuals’ psychological resources that support adaptive functioning (Wang et al., 2025).

The Broaden-and-Build Theory further suggests that positive emotional states expand individuals’ cognitive and behavioral repertoires, enabling them to develop enduring personal resources that support long-term growth and adaptation (Fredrickson, 2001). As emotional burdens are alleviated, students become more capable of engaging in self-reflection, exploring new perspectives, and pursuing personal development goals (Yavuz Sercekman, 2024).

Research in art and well-being has similarly argued that emotional recovery facilitates deeper self-awareness and self-understanding. Malchiodi (2006) emphasized that emotional expression and recovery through artistic activities promote personal insight and self-discovery. Van Den Akker (2014) further suggested that heightened awareness of sensory and emotional experiences supports self-realization and psychological development. Within outdoor landscape sketching courses, emotional healing may provide students with opportunities to reflect on their experiences, strengthen their sense of self-worth, and cultivate greater personal awareness. Consequently, emotional healing functions not only as a desirable psychological outcome of outdoor landscape sketching experiences, but also as a foundation for subsequent personal growth.

Therefore, the following hypothesis is proposed:

*H6*: Students’ emotional healing positively influences their perceived personal growth.

## 3 Method

### 3.1 Conceptual model

This study proposes a conceptual model based on an extension of the Stimulus-Organism-Response (SOR) framework. Originally developed by Mehrabian and Russell (1974), the SOR framework explains how environmental stimuli influence individuals’ internal psychological states, which subsequently shape their psychological and behavioral responses. The framework has been widely applied in environmental psychology, consumer behavior, and educational research to examine the relationships between environmental experiences and individual outcomes (Bigne et al., 2020; Vieira, 2013).

However, the traditional SOR framework primarily focuses on immediate psychological and behavioral responses. In the context of outdoor landscape sketching, students’ experiences may extend beyond emotional reactions and be associated with broader developmental outcomes. To capture this process, the present study extends the traditional SOR framework by incorporating a Consequences (C) dimension, resulting in a Stimulus–Organism–Response–Consequences framework. Within the proposed framework, emotions experienced in natural environments (EN) and cultural environments (EC) are conceptualized as environmental stimuli (S). Artistic creation (AC) represents the organismic process (O), reflecting students’ cognitive, emotional, and expressive engagement with environmental experiences during outdoor landscape sketching activities (Kaplan and Kaplan, 1989). Perceived emotional healing (PEH) is conceptualized as a psychological response (R), reflecting students’ perceived emotional improvement, emotional regulation, and positive psychological experiences associated with participation in outdoor landscape sketching activities (Clatworthy et al., 2013). Perceived personal growth (PPG) is positioned as a developmental consequence (C) representing students’ self-reported experiences of self-awareness, personal development, and psychological enrichment associated with engagement in outdoor landscape sketching courses.

**Figure 1:**
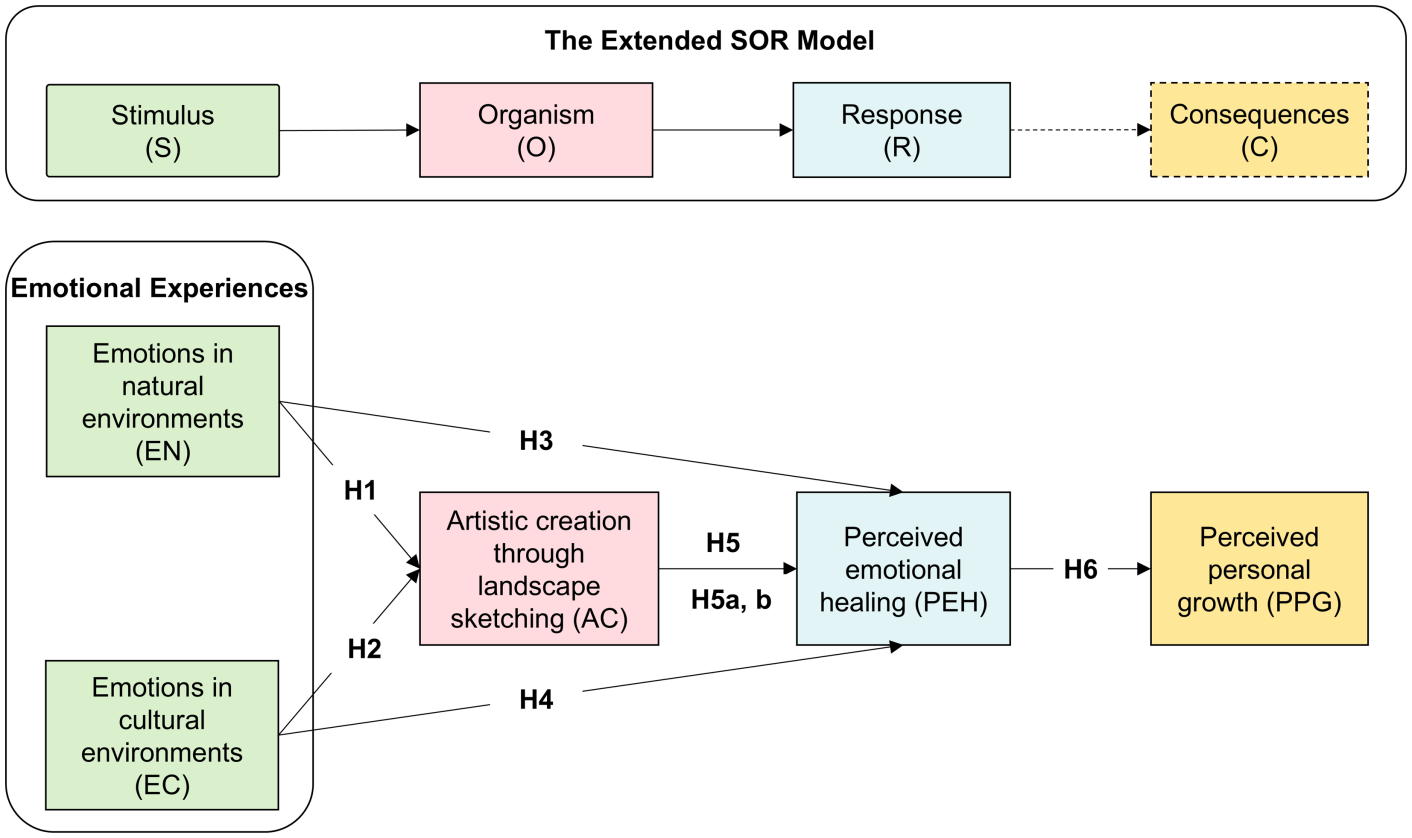
Proposed conceptual model based on the extended SOR framework. The dashed box represents the extended consequences (C) component beyond the traditional SOR process.

This framework provides a structured approach for investigating the psychological and educational value of landscape sketching in higher education settings. It also offers a theoretical foundation for understanding how nature-based and arts-based educational experiences may support students’ well-being and personal development (Li and Qi, 2025). Figure 1 presents the proposed conceptual model and hypothesized relationships among the study constructs.

Given the cross-sectional and self-reported nature of the data, the proposed model is intended to examine associations among students’ perceived experiences rather than establish causal relationships or temporal ordering among the constructs.

### 3.2 Instrument development and measures

Data were collected using a structured questionnaire designed to assess students’ environmental experiences, artistic creation, perceived emotional healing, and perceived personal growth during outdoor landscape sketching courses. The questionnaire items were developed based on relevant theoretical constructs and empirical findings reported in previous studies and were subsequently refined to fit the educational context of outdoor landscape sketching (Table 1).

**Table 1:** Initial measurement items for the five study constructs.

Construct	Dimension	No.	Items	References
Stimulus	Emotions in natural environments	EN1	The natural landscapes in the outdoor Landscape Sketching course made me feel calm and relaxed.	Kaplan and Kaplan (1989), Pan et al. (2024)
		EN2	The natural landscapes in the outdoor Landscape Sketching course made me feel happy and joyful.	
		EN3	The natural landscapes in the outdoor Landscape Sketching course helped relieve my anxiety.	
		EN4	The vast natural landscapes in the outdoor Landscape Sketching course evoked a sense of awe in me.	
	Emotions in cultural environments	EC1	Visiting museums during the outdoor Landscape Sketching course gave me a sense of happiness in a peaceful setting.	Sang (2024), Tian et al. (2024)
		EC2	Visiting digital museums based on traditional culture during the outdoor Landscape Sketching course strengthened my sense of nostalgia.	
		EC3	Visiting cultural sites during the outdoor Landscape Sketching course gave me a sense of respect for nostalgic culture.	
		EC4	The cultural landscapes in the outdoor Landscape Sketching course evoked my sense of cultural identity.	
		EC5^*^	Visiting modern architectural landscapes, such as skyscrapers, during the outdoor Landscape Sketching course gave me a sense of excitement.	
Organism	Artistic creation through landscape sketching	AC1	In the creative practice of the outdoor Landscape Sketching course, I have been using colors more freely and boldly.	Wango (2022)
		AC2	In the creative practice of the outdoor Landscape Sketching course, I am able to observe environmental details more keenly and vividly present them in my artwork.	
		AC3	In the creative practice of the outdoor Landscape Sketching course, I am able to more realistically portray the texture characteristics of natural elements (such as mountains, water, and trees).	
		AC4	In the creative practice of the outdoor Landscape Sketching course, I am able to better apply perspective techniques in composition.	
Response	Perceived emotional healing	PEH1	After the artistic creation in the outdoor Landscape Sketching course, I am able to release emotions and achieve emotional catharsis.	Csikszentmihalyi (1990), Wango (2022), Lancaster (2017)
		PEH2	During the outdoor Landscape Sketching course, immersing myself in artistic creation brings me a sense of pleasure and joy.	
		PEH3	During the outdoor Landscape Sketching course, using different painting tools helps alleviate stress and brings a sense of inner peace.	
		PEH4	During the outdoor Landscape Sketching course, I am able to sense the surrounding energy.	
		PEH5	During the outdoor Landscape Sketching course, I feel encouraged to explore artistic expression with greater boldness.	
Consequences	Perceived personal growth	PPG1	The emotional catharsis brought by artistic creation can enhance my self-awareness.	Stuckey and Nobel (2010), Csikszentmihalyi (1990)
		PPG2	The rich emotional experiences brought by artistic creation help me gain a deeper understanding of myself.	
		PPG3	The sense of achievement gained through artistic creation enhances my self-worth.	
		PPG4	Experiencing flow during artistic creation boosts my intrinsic motivation.	

^*^Indicates that EC5 was removed from subsequent analyses following exploratory factor analysis.

To establish content validity, the preliminary questionnaire was reviewed by three experts specializing in environmental design, art education, and educational psychology. The experts evaluated the items in terms of conceptual relevance, representativeness, clarity, and contextual appropriateness. Based on their feedback, several items were revised to improve wording clarity and alignment with the study context.

Because several source studies and measurement items were originally reported in English, a translation–back translation procedure was employed to ensure linguistic and conceptual equivalence (Brislin, 1970). The items were translated into Chinese and independently back-translated into English by bilingual researchers, with any discrepancies resolved through discussion. The final Chinese version was reviewed by the expert panel and pilot-tested with eight students who had previous landscape sketching experience, resulting in minor wording refinements before formal data collection.

The questionnaire comprised two sections. The first section collected participants’ demographic information, including gender, age, academic year, and prior landscape sketching experience. The second section initially consisted of 22 measurement items assessing the five study constructs using a five-point Likert scale ranging from 1 (“strongly disagree”) to 5 (“strongly agree”).

As listed in Table 1, the initial measurement items were adapted from previously validated scales and modified to fit the context of landscape sketching courses. Emotions in Natural Environments (EN) were measured using four items assessing students’ emotional responses to natural landscapes encountered during landscape sketching activities. Emotions in Cultural Environments (EC) were measured using five items capturing emotional experiences associated with culturally meaningful environments, including traditional culture, historical heritage, and cultural identity. Artistic Creation (AC) was measured using four items evaluating students’ perceived creative engagement and artistic expression during the sketching process. Perceived Emotional Healing (PEH) was measured using five items assessing emotional improvement, emotional regulation, psychological comfort, and positive emotional experiences associated with landscape sketching. Perceived Personal Growth (PPG) was measured using four items examining perceived self-development, enhanced self-awareness, confidence, and personal enrichment.

Following exploratory factor analysis, one item from the Emotions in Cultural Environments construct (EC5) was removed because it demonstrated insufficient statistical and conceptual alignment with the remaining indicators. The final measurement instrument therefore comprised 21 items across the five constructs. The retained item counts per construct are as follows: EN = 4, EC = 4, AC = 4, PEH = 5, and PPG = 4.

### 3.3 Participants and sampling

This research adopted a purposive sampling technique to recruit undergraduate students who had completed or were currently enrolled in outdoor landscape sketching courses. All participating students received outdoor landscape sketching training as part of their design-related curriculum. Participants were drawn from three higher education institutions in China that offered comparable landscape sketching curricula within art and design programs. Although implemented at different institutions, the courses were characterized by similar learning objectives, experiential sketching activities, and assessment requirements, providing a broadly comparable educational context for the present study.

Data were collected through an online questionnaire administered between April 1 and April 15, 2025. The questionnaire was distributed electronically via WeChat and WeCom. As part of the eligibility screening process, respondents who self-reported having received a formal psychiatric diagnosis or psychotherapy within the previous 3 months were excluded from the study. Screening was conducted through self-report questions included in the demographic section of the questionnaire, and no medical records were requested or collected.

A total of 363 questionnaires were returned. Following eligibility screening and data quality checks, 13 responses were excluded: four due to ineligibility, six due to incomplete questionnaires and three due to duplicate submissions. The final sample consisted of 350 valid responses, which were used in all subsequent analyses.

This study was approved by the Ethics Committee of the first author’s affiliated institution (Approval No. SDA-EC-2025-S-001). Participation was voluntary, informed consent was obtained from all respondents prior to data collection, and all questionnaire responses were collected anonymously. No personally identifiable information was recorded, and all results are reported in aggregate form.

### 3.4 Statistical analysis

Data analysis was conducted in three stages. First, descriptive statistics were calculated to summarize the characteristics of the sample and the distribution of the study variables. Exploratory factor analysis (EFA) was then performed using SPSS 26.0 to examine the underlying factor structure of the questionnaire and assess the suitability of the measurement items.

Second, Partial Least Squares Structural Equation Modeling (PLS-SEM) was performed using SmartPLS 4.1 to evaluate the proposed conceptual model. PLS-SEM was considered appropriate because the study involved multiple latent constructs, mediation pathways, and an exploratory research framework aimed at examining predictive relationships among variables. Following recommended procedures, the measurement model was first assessed in terms of indicator reliability, internal consistency reliability, convergent validity, and discriminant validity. Indicator reliability was evaluated using outer loadings, while Cronbach’s alpha (CA) and composite reliability (CR) were used to assess internal consistency. Convergent validity was assessed using average variance extracted (AVE). Discriminant validity was evaluated using both the Fornell–Larcker criterion and the heterotrait–monotrait ratio (HTMT). Indicator-level variance inflation factor (VIF) values were examined to assess potential multicollinearity among measurement indicators.

Third, the structural model was assessed by examining collinearity, path coefficients, effect sizes (*f^2^*), coefficients of determination (*R^2^*), and predictive relevance (*Q^2^*_predict_). Structural-level variance inflation factor (VIF) values were first evaluated to assess collinearity among predictor constructs. Bootstrapping with 5,000 resamples was performed to estimate standard errors, 95% confidence intervals, and significance levels for direct, indirect, and total effects. Mediation effects were evaluated by examining the significance and confidence intervals of indirect effects obtained through bootstrapping. Statistical significance was evaluated at *p* < 0.05.

## 4 Results

### 4.1 Participant characteristics

The final sample consisted of 350 university students enrolled in art and design programs. As shown in Table 2, 72.3% of the participants were female (*n* = 253), whereas 27.7% were male (*n* = 97). Regarding academic level, the majority of participants were juniors (54.2%, *n* = 190), followed by sophomores (44.6%, *n* = 156), and a small proportion were seniors (1.2%, *n* = 4). In terms of academic major, Environmental Design students accounted for the largest proportion of the sample (32.0%, *n* = 112), followed by Product Design (21.7%, *n* = 76), Painting (20.6%, *n* = 72), Visual Communication Design (19.1%, *n* = 67), Drama (5.4%, *n* = 19), and Animation (1.1%, *n* = 4). As for prior outdoor landscape sketching experience, 43.1% of participants (*n* = 151) reported frequent participation in outdoor landscape sketching, while the majority (52.6%, *n* = 184) reported occasional participation. Only a small proportion reported rare participation (4.0%, *n* = 14) or no prior participation (0.3%, *n* = 1).

**Table 2:** Participant characteristics.

Demographic factor	Categories	Frequency	Valid percent	Cumulative percent
Gender	Male	97	27.7	27.7
	Female	253	72.3	100
Grade	Sophomore	156	44.6	44.6
	Junior	190	54.2	98.8
	Senior	4	1.2	100
Major	Environmental design	112	32.0	32.0
	Visual communication design	67	19.1	51.1
	Product design	76	21.7	72.9
	Animation	4	1.1	74.0
	Painting	72	20.6	94.6
	Drama	19	5.4	100.0
Experience	Frequently participate in outdoor landscape sketching	151	43.1	43.1
	Occasionally participate in outdoor landscape sketching	184	52.6	95.7
	Rarely participate in outdoor landscape sketching	14	4.0	99.7
	Never participate in outdoor landscape sketching	1	0.3	100
Total		350	100	

### 4.2 Exploratory factor analysis

Prior to evaluating the proposed conceptual framework, exploratory factor analysis (EFA) was conducted to examine the underlying structure of the measurement instrument. The suitability of the data for factor analysis was confirmed by a Kaiser–Meyer–Olkin (KMO) value of 0.968 and a significant Bartlett’s test of sphericity (*p* < 0.001), indicating that the correlation matrix was appropriate for factor extraction.

EFA was performed using principal component extraction with Varimax rotation. Five components with eigenvalues greater than 1.0 were extracted, explaining 81.53% of the total variance. As presented in Table 3, the extracted conponent structure was generally consistent with the proposed conceptual dimensions, and all retained indicators loaded primarily on their intended constructs.

**Table 3:** Rotated component matrix.

Item	Component				
	1	2	3	4	5
EN1	0.240	0.408	**0.733**	0.158	0.297
EN2	0.312	0.380	**0.755**	0.164	0.223
EN3	0.217	0.295	**0.753**	0.332	0.216
EN4	0.217	0.413	**0.614**	0.320	0.221
EC1	0.201	**0.702**	0.379	0.266	0.198
EC2	0.258	**0.713**	0.316	0.254	0.279
EC3	0.212	**0.759**	0.326	0.275	0.194
EC4	0.311	**0.719**	0.319	0.236	0.233
AC1	0.402	0.311	0.293	**0.645**	0.288
AC2	0.317	0.369	0.233	**0.642**	0.383
AC3	0.389	0.361	0.258	**0.647**	0.290
AC4	0.321	0.325	0.260	**0.695**	0.300
PEH1	**0.591**	0.174	0.387	0.486	0.257
PEH2	**0.623**	0.207	0.334	0.458	0.231
PEH3	**0.709**	0.251	0.245	0.293	0.394
PEH4	**0.697**	0.310	0.172	0.360	0.301
PEH5	**0.705**	0.333	0.276	0.197	0.333
PPG1	0.430	0.316	0.274	0.389	**0.586**
PPG2	0.357	0.261	0.320	0.302	**0.702**
PPG3	0.424	0.295	0.243	0.350	**0.643**
PPG4	0.364	0.284	0.326	0.299	**0.675**

Exploratory factor analysis (EFA) was performed using principal component extraction with Varimax rotation and Kaiser normalization. Five components with eigenvalues greater than 1.0 were extracted, accounting for 81.525% of the total variance. Factor loadings greater than 0.50 are shown in bold. The rotation converged in eight iterations. EN, emotions in natural environments; EC, emotions in cultural environments; AC, artistic creation through landscape sketching; PEH, perceived emotional healing; PPG, perceived personal growth.

One item from the Emotions in Cultural Environments construct (EC5) was removed because its factor loading fell below the recommended threshold and it demonstrated weaker conceptual alignment with the remaining indicators. From a theoretical perspective, EC1–EC4 primarily reflect emotional experiences associated with cultural heritage, historical memory, and cultural identity, whereas EC5 focused on excitement generated by contemporary urban architectural landscapes. This item appears to capture novelty and environmental stimulation rather than culturally meaningful emotional experiences. Therefore, removing EC5 improved the conceptual coherence of the cultural environment construct while preserving its theoretical focus on culturally meaningful environmental experiences. Following its removal, all remaining indicators exhibited satisfactory factor loadings above 0.50 on their respective constructs.

### 4.3 Measurement model assessment

The measurement model was evaluated in terms of indicator reliability, internal consistency reliability, convergent validity, and discriminant validity. As shown in Table 4, all indicator loadings exceeded the recommended threshold of 0.70, and all indicator-level VIF values were below 5.0, indicating satisfactory indicator reliability and the absence of problematic multicollinearity (Hair et al., 2022).

**Table 4:** Measurement model assessment.

Constructs	Indicators	Outer loadings	Indicator VIF	Cronbach’s alpha	CR	AVE
AC	AC1	0.924	3.913	0.944	0.960	0.856
	AC2	0.924	3.947			
	AC3	0.930	4.214			
	AC4	0.923	3.924			
PEH	PEH1	0.903	3.258	0.942	0.955	0.811
	PEH2	0.893	3.567			
	PEH3	0.922	3.806			
	PEH4	0.897	3.857			
	PEH5	0.887	4.622			
EC	EC1	0.891	4.875	0.926	0.947	0.818
	EC2	0.908	3.415			
	EC3	0.909	2.551			
	EC4	0.910	4.152			
EN	EN1	0.929	3.879	0.933	0.952	0.833
	EN2	0.936	4.507			
	EN3	0.911	3.577			
	EN4	0.875	3.385			
PPG	PPG1	0.934	4.426	0.948	0.962	0.865
	PPG2	0.929	4.240			
	PPG3	0.931	4.314			
	PPG4	0.925	4.046			

AC, artistic creation through landscape sketching; PEH, perceived emotional healing; EC, emotions in cultural environments; EN, emotions in natural environments; PPG, perceived personal growth. AVE, average variance extracted; CR, composite reliability. Recommended thresholds (Hair et al., 2022) are outer loadings ≥ 0.70, indicator VIF < 5.0, Cronbach’s alpha ≥ 0.70, CR ≥ 0.70, and AVE ≥ 0.50.

The constructs also demonstrated satisfactory internal consistency reliability and convergent validity. Cronbach’s alpha values ranged from 0.926 to 0.948, while composite reliability values ranged from 0.947 to 0.962. In addition, the average variance extracted (AVE) values ranged from 0.811 to 0.865, exceeding the recommended threshold of 0.50.

Discriminant validity was assessed using both the Fornell–Larcker criterion and the heterotrait–monotrait ratio (HTMT). As presented in Table 5, the square root of the AVE for each construct exceeded its correlations with all other constructs, supporting discriminant validity according to the Fornell–Larcker criterion. The HTMT values ranged from 0.796 to 0.930. Although several HTMT values exceeded the conservative threshold of 0.85, all bootstrap 95% confidence intervals remained below 1.00, Therefore, indicating acceptable discriminant validity among the study constructs (Henseler et al., 2015). Overall, the measurement model demonstrated satisfactory psychometric properties and was considered suitable for subsequent structural model evaluation.

**Table 5:** Assessment of discriminant validity assessment.

Constructs	AC	EC	EN	PEH	PPG
Panel A: Fornell-Larcker criterion					
AC	**0.925**				
EC	0.785	**0.905**			
EN	0.760	0.827	**0.913**		
PEH	0.871	0.744	0.754	**0.900**	
PPG	0.854	0.761	0.764	0.879	**0.930**
Panel B: Heterotrait-Monotrait Ratios (HTMT) and associated confidence intervals					
AC	—				
EC	0.839 (0.780–0.894)	—			
EN	0.810 (0.740–0.870)	0.890 (0.843–0.932)	—		
PEH	0.924 (0.884–0.956)	0.796 (0.734–0.854)	0.805 (0.735–0.868)	—	
PPG	0.902 (0.856–0.942)	0.811 (0.748–0.867)	0.813 (0.751–0.871)	0.930 (0.891–0.964)	—

Panel A reports the Fornell–Larcker criterion, where diagonal elements in bold represent the square roots of the average variance extracted (AVE) and off-diagonal elements represent inter-construct correlations. Panel B reports the heterotrait–monotrait ratio of correlations (HTMT), with the corresponding 95% bootstrap confidence intervals presented in parentheses below each HTMT value. AC, artistic creation through landscape sketching; EC, emotions in cultural environments; EN, emotions in natural environments; PEH, perceived emotional healing; PPG, perceived personal growth.

### 4.4 Structural model results

**Figure 2:**
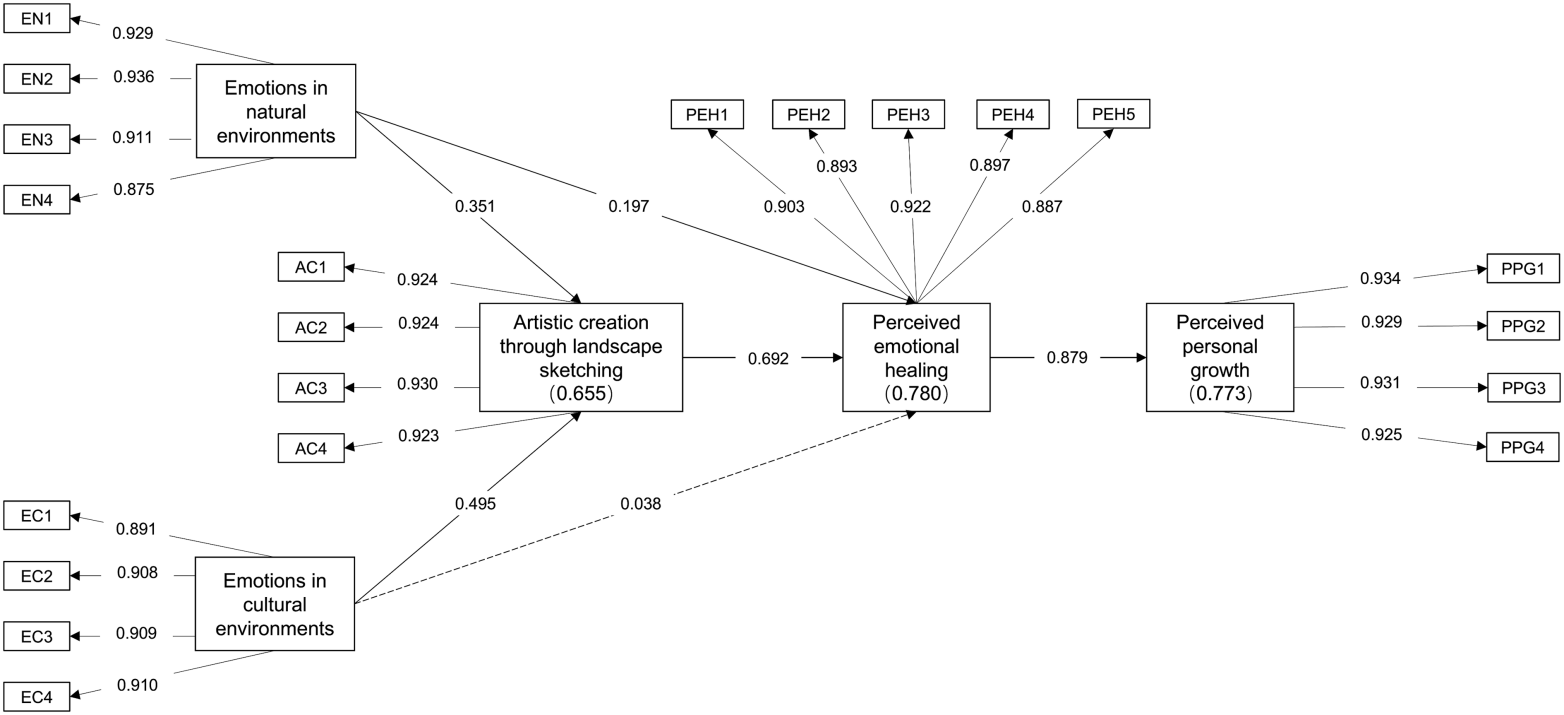
Results of the PLS-SEM Analysis of the Structural Model. Values on the paths represent standardized path coefficients (*β*), and values in parentheses indicate the coefficient of determination (*R^2^*) for endogenous constructs. Solid lines represent significant relationships (*p* < 0.05), whereas dashed lines represent non-significant relationships (*p* ≥ 0.05). AC, artistic creation through landscape sketching; EC, emotions in cultural environments; EN, emotions in natural environments; PEH, perceived emotional healing; PPG, perceived personal growth.

The structural model was evaluated by examining the hypothesized relationships among environmental emotions, artistic creation, perceived emotional healing, and perceived personal growth. The estimated structural model with standardized path coefficients is presented in Figure 2, and detailed results are reported in Table 6. Furthermore, all structural-level VIF values were below the recommended threshold of 5.0, indicating the absence of problematic collinearity among the predictor constructs.

**Table 6:** Direct effects and hypothesis testing results.

Hypothesis	Path	*β*	*t*	*p*	95% CI LL	95% CI UL	VIF	*f^2^*	Results
H1	EN → AC	0.351	4.057	<0.001	0.180	0.518	3.158	0.113	Supported
H2	EC → AC	0.495	6.021	<0.001	0.330	0.654	3.158	0.225	Supported
H3	EN → PEH	0.197	3.057	0.002	0.071	0.321	3.515	0.050	Supported
H4	EC → PEH	0.038	0.680	0.497	−0.070	0.152	3.868	0.002	Rejected
H5	AC → PEH	0.692	11.085	<0.001	0.556	0.801	2.899	0.750	Supported
H6	PEH → PPG	0.879	46.367	<0.001	0.841	0.914	1.000	3.412	Supported

*β*, standardized path coefficient; CI, confidence interval; LL, lower limit; UL, upper limit; VIF, variance inflation factor (structural model); *f^2^*, effect size. Confidence intervals were estimated using bootstrapping (5,000 resamples). Relationships were considered statistically significant when *p* < 0.05 and the 95% confidence interval did not include zero. Following Hair et al. (2022), *f^2^* values of 0.02, 0.15, and 0.35 indicate small, medium, and large effects, respectively. EN, emotions in natural environments; EC, emotions in cultural environments; AC, artistic creation through landscape sketching; PEH, perceived emotional healing; PPG, perceived personal growth.

The results showed that emotions in natural environments (EN) had a significant positive effect on artistic creation through landscape sketching (*β* = 0.351, *p* < 0.001), supporting H1. Similarly, emotions in cultural environments (EC) were positively associated with artistic creation (*β* = 0.495, *p* < 0.001), supporting H2. The effect of emotions in cultural environments was stronger than that of emotions in natural environments.

Regarding perceived emotional healing, both emotions in natural environments (*β* = 0.197, *p* = 0.002) and artistic creation (*β* = 0.692, *p* < 0.001) exerted significant positive effects, supporting H3 and H5, respectively. In contrast, the direct effect of emotions in cultural environments on perceived emotional healing was not statistically significant (*β* = 0.038, *p* = 0.497), leading to the rejection of H4.

Finally, perceived emotional healing exerted a strong positive effect on perceived personal growth (*β* = 0.879, *p* < 0.001), supporting H6. Among all hypothesized relationships, this path demonstrated both the largest standardized coefficient and the strongest effect size (*f^2^*), underscoring the pivotal role of perceived emotional healing in students’ perceived personal growth during outdoor landscape sketching courses.

### 4.5 Mediation analysis

To further examine the mediating role of artistic creation through outdoor landscape sketching in the relationships between environmental emotions and perceived emotional healing, a bootstrapping procedure with 5,000 resamples and bias-corrected 95% confidence intervals was conducted. As shown in Table 7, both indirect effects were statistically significant because their 95% confidence intervals did not include zero.

**Table 7:** Mediation analysis results.

Hypothesis	Indirect path	Indirect effect *β*	Total effect *β*	*t*	*p*	95% CI LL	95% CI UL	Mediation type
H5a	EN → AC → PEH	0.243	0.439	3.930	< 0.001	0.124	0.367	Partial mediation
H5b	EC → AC → PEH	0.342	0.381	5.208	< 0.001	0.217	0.477	Indirect-only mediation

Indirect effects were assessed using bootstrapping with 5,000 resamples. Mediation was considered significant when the 95% bootstrap confidence interval did not include zero. Partial mediation indicates that both the direct and indirect effects are significant, whereas indirect-only mediation indicates that the indirect effect is significant while the direct effect is not. AC, artistic creation through landscape sketching; PEH, perceived emotional healing; EN, emotions in natural environments; EC, emotions in cultural environments.

Specifically, artistic creation through outdoor landscape sketching significantly mediated the relationship between emotions in natural environments and perceived emotional healing (*β* = 0.243, *t* = 3.930, *p* < 0.001, 95% CI [0.124, 0.367]), supporting H5a. The total effect of emotions in natural environments on perceived emotional healing was 0.439. Because both the direct effect (EN → PEH; *β* = 0.197, *p* = 0.002) and the indirect effect (EN → AC → PEH) were statistically significant, this relationship was classified as complementary partial mediation according to Zhao et al. (2010). These findings suggest that emotions in natural environments contribute to perceived emotional healing both directly and indirectly through enhanced artistic creation.

Similarly, artistic creation through landscape sketching significantly mediated the relationship between emotions in cultural environments and perceived emotional healing (*β* = 0.342, *t* = 5.208, *p* < 0.001, 95% CI [0.217, 0.477]), supporting H5b. The total effect of emotions in cultural environments on perceived emotional healing was 0.381. Because the indirect effect was significant whereas the direct effect (EC → PEH; *β* = 0.038, *p* = 0.497) was not, this relationship was classified as indirect-only mediation according to Zhao et al. (2010). These findings indicate that the emotional benefits associated with cultural environments are realized primarily through students’ engagement in artistic creation rather than through a direct effect on perceived emotional healing.

Overall, the indirect effect associated with emotions in cultural environments was stronger than that associated with emotions in natural environments, suggesting that artistic creation plays a particularly important role in transforming culturally meaningful emotional experiences into perceived emotional healing. These findings highlight artistic creation through landscape sketching as an important mechanism linking students’ emotional experiences in outdoor environments with perceived emotional healing.

### 4.6 Explanatory power and predictive relevance

The explanatory power and predictive relevance of the proposed conceptual framework were evaluated using *R^2^* and *Q^2^*_predict_ values. The results are summarized in Table 8.

**Table 8:** Explanatory power and predictive relevance of the conceptual framework.

Endogenous construct	*R^2^*	*Q^2^*_predict_
AC	0.655	0.644
PEH	0.780	0.605
PPG	0.773	0.620

*R^2^*, coefficient of determination; *Q^2^*_predict_ values greater than zero indicate predictive relevance. AC, artistic creation through landscape sketching; PEH, perceived emotional healing; PPG, perceived personal growth.

The model demonstrated substantial explanatory power, with *R^2^* values of 0.655 for Artistic Creation (AC), 0.780 for Perceived Emotional Healing (PEH), and 0.773 for Perceived Personal Growth (PPG). These results indicate that the model explained a considerable proportion of the variance in the endogenous constructs.

Furthermore, all endogenous constructs exhibited positive *Q^2^*_predict_ values (AC = 0.644, PEH = 0.605, and PPG = 0.620), indicating satisfactory out-of-sample predictive relevance. Overall, these findings demonstrate that the proposed conceptual framework possesses strong explanatory power and adequate predictive performance.

## 5 Discussion

### 5.1 Environmental emotions and artistic creation

This study examined the associations between environmental emotions and artistic creation within outdoor landscape sketching courses. The findings suggest that emotions associated with both natural and cultural environments were positively related to artistic creation, indicating that different environmental contexts may provide distinct emotional and aesthetic resources that support students’ creative engagement.

Students reported positive emotional experiences in both natural and cultural environments, although the characteristics of these experiences appeared to differ. Emotions in natural environments were commonly associated with calmness, awe, and joy, whereas cultural environments more often evoked feelings related to cultural identity, historical connection, and nostalgia. These findings are broadly consistent with previous studies suggesting that natural environments promote emotional restoration (Bratman et al., 2019; Kaplan and Kaplan, 1989; White et al., 2021), while cultural environments evoke symbolic meaning and emotional resonance through cultural heritage and collective memory (Sang, 2024; Taylor and Lennon, 2012).

Both types of emotional experiences were positively associated with artistic creation, suggesting that emotional engagement may provide an important psychological foundation for creative practice. During outdoor landscape sketching, students do not simply reproduce visual scenes but actively integrate environmental perceptions, emotional experiences, and aesthetic understanding into artistic expression. Positive emotional experiences may therefore encourage sustained observation, deeper environmental engagement, and more reflective creative processes.

Notably, emotions associated with cultural environments exhibited a larger effect on artistic creation (*f^2^* = 0.225) than natural environments (*f^2^* = 0.113). One possible explanation is that cultural environments may provide not only aesthetic stimuli but also historical, social, and symbolic meanings that encourage interpretation and reflection. Compared with natural environments, cultural settings may therefore offer richer sources of creative inspiration by engaging students in processes of cultural understanding and meaning-making.

Overall, these findings suggest that natural and cultural environments may support artistic creation through partially different psychological processes. While natural environments appear to facilitate creativity primarily through positive emotional experiences, cultural environments may additionally promote artistic creation by stimulating symbolic interpretation and cultural reflection.

### 5.2 The mediating role of artistic creation

One of the most notable findings of this study is that emotions associated with cultural environments were not directly related to perceived emotional healing, but demonstrated a significant indirect effect through artistic creation. This pattern suggests that natural and cultural environments may contribute to students’ perceived psychological experiences through different pathways.

A plausible explanation is that cultural environments evoke emotional experiences that are primarily symbolic and interpretive rather than immediately restorative. Unlike natural environments, which are often associated with calmness and emotional restoration, cultural environments may stimulate reflection on cultural identity, historical continuity, and collective memory. These experiences may not directly contribute to perceived emotional healing but instead provide meaningful content that is transformed into personally relevant experiences through artistic creation.

Outdoor landscape sketching provides students with opportunities to observe, interpret, and visually express these environmental experiences. Rather than simply reproducing physical scenes, students actively construct meaning by integrating emotional responses, aesthetic perception, and cultural understanding into the creative process. Through this process of artistic meaning-making, emotional experiences associated with cultural environments may become personally meaningful through creative experiences, thereby contributing to perceived emotional healing. This interpretation is consistent with the significant indirect effect observed in the present study (EC → AC → PEH; *β* = 0.342, *p* < 0.001).

More broadly, the findings highlight artistic creation as a critical psychological process linking environmental emotions with perceived emotional healing. Through creative practice, students externalize emotions, reflect on their environmental experiences, and construct personally meaningful interpretations. Accordingly, artistic creation is theoretically expected to be closely associated with perceived emotional healing and perceived personal growth, as it represents the core educational process through which environmental experiences are transformed into perceived psychological benefits. These relatively strong associations are therefore theoretically plausible while the constructs remain conceptually distinguishable within the proposed framework.

Taken together, these findings suggest that natural and cultural environments may support students’ perceived emotional well-being through partially different psychological pathways. Whereas emotions associated with natural environments appear to be more directly related to perceived emotional healing, emotions associated with cultural environments may exert their influence primarily through artistic creation and symbolic meaning-making. This distinction extends previous research by demonstrating that different environmental contexts may contribute to perceived psychological benefits through different mechanisms within an arts-based educational setting.

### 5.3 Perceived emotional healing and personal growth

The findings further suggest that perceived emotional healing was positively associated with perceived personal growth, indicating that students who reported greater emotional benefits from outdoor landscape sketching also tended to report stronger perceptions of personal development. This finding extends previous research by suggesting that the value of outdoor landscape sketching may lie not only in supporting positive emotional experiences but also in fostering broader educational and developmental outcomes.

One possible explanation is that emotional healing may provide psychological conditions that enable individuals to engage more positively with learning experiences and personal development. When students experience greater emotional balance, inner calmness, or emotional relief, they may be more willing to explore new ideas, persist in challenging tasks, and engage in reflective learning (Kaimal et al., 2016). These psychological changes may contribute to stronger perceptions of confidence, self-awareness, and personal growth. This interpretation is broadly consistent with previous studies suggesting that positive emotional experiences are closely associated with self-development and psychological well-being (Malchiodi, 2006; Stuckey and Nobel, 2010).

Nevertheless, the strong association between perceived emotional healing and perceived personal growth should be interpreted cautiously. These constructs represent different stages of students’ educational experiences—immediate psychological responses and perceived developmental outcomes—and are therefore theoretically expected to be closely related. At the same time, because both constructs were measured through self-reported perceptions at a single time point and exhibited relatively high intercorrelations, the observed relationship may partly reflect conceptual proximity between positive emotional experiences and perceptions of personal development. Future longitudinal, experimental, and multi-method research is needed to further examine the conceptual distinctiveness and temporal relationships between these constructs.

## 6 Implications

This study offers both theoretical and practical implications for understanding the role of outdoor landscape sketching in higher art education, especially in fostering students’ emotional well-being and holistic development.

### 6.1 Theoretical implications

Compared with the traditional SOR framework, the proposed extended SOR framework incorporates perceived emotional healing and perceived personal growth as additional outcome dimensions within an arts-based educational context. Rather than conceptualizing environmental experiences as a single pathway leading to psychological outcomes, the framework distinguishes the potentially different roles of natural and cultural environments in shaping students’ emotional and creative experiences. Furthermore, the findings identify artistic creation as an important mechanism linking environmental experiences with perceived psychological benefits, thereby extending the application of the SOR framework to arts-based educational contexts. Accordingly, the proposed framework provides a theoretical foundation for future research integrating art education, environmental psychology, and arts-based well-being.

### 6.2 Practical implications

At the philosophical level, the findings suggest moving beyond purely skill-centered curricula toward a more holistic educational framework that integrates artistic proficiency with emotional development and personal growth. By situating creative practice within natural and cultural environments, students not only cultivate technical and aesthetic competencies but also engage in meaningful emotional experiences that encourage self-regulation, emotional awareness, and personal insight. Accordingly, outdoor sketching courses may serve as valuable arts-based learning experiences that support both artistic development and students’ perceived well-being.

In terms of pedagogical strategy, the research supports a three-phase instructional approach of “Observation–Expression–Reflection.” This model encourages students to move beyond passive technical reproduction by engaging in deep perceptual immersion, open-ended artistic exploration, and metacognitive reflection. In particular, the reflection phase, which involves peer feedback, dialogic teacher-student evaluation, and reflective writing, may facilitate the internalization of aesthetic experiences and the development of higher-order thinking skills. The findings further suggest that integrating structured reflective activities into outdoor sketching courses may enhance students’ engagement with both the environment and the creative process.

Practically, the conceptual model may have interdisciplinary relevance across several fields. In higher education, it provides a conceptual basis for incorporating experiential and arts-based learning activities into student well-being initiatives and creativity development programs. In student support settings, the findings highlight the potential value of arts-based educational activities as complementary resources for promoting emotional expression, reflection, and engagement. The findings further suggest that natural and cultural environments may be intentionally incorporated into outdoor sketching activities in different ways, as they appear to contribute to students’ experiences through distinct psychological pathways. In urban planning and environmental design, the framework may inspire the development of culturally meaningful and aesthetically engaging public spaces that encourage creative interaction, environmental appreciation, and community participation.

## 7 Conclusion and limitations

### 7.1 Conclusion

This study applied the Stimulus-Organism-Response (SOR) framework to examine the associations among environmental emotions, artistic creation, perceived emotional healing, and perceived personal growth within outdoor landscape sketching courses. Based on empirical data from 350 undergraduate art and design students in China, the study proposed and tested an extended SOR framework by incorporating a Consequences (C) dimension to examine these relationships within an arts-based educational context.

The findings suggest that artistic creation was positively associated with perceived emotional healing, which in turn was positively associated with perceived personal growth. Furthermore, the results indicate that natural and cultural environments may contribute to students’ experiences through distinct psychological pathways. Whereas emotions in natural environments were directly associated with perceived emotional healing, the influence of cultural environments appeared to operate primarily through artistic creation, highlighting the role of creative meaning-making in transforming culturally experiences into perceived psychological benefits.

The findings advance current understanding of the psychological value of outdoor landscape sketching by demonstrating how environmental experiences may be associated with perceived psychological benefits through artistic creation. The incorporation of the Consequences dimension offers a conceptual foundation for future research in arts-based education, environmental psychology, and arts-based well-being by distinguishing the psychological pathways through which natural and cultural environments operate. Practically, the findings support the integration of outdoor landscape sketching into higher art education as an experiential learning approach that may foster students’ emotional well-being and personal development. Future longitudinal, experimental, and multi-method research is needed to further examine the temporal relationships, underlying mechanisms, and generalizability of the proposed framework.

### 7.2 Limitations

Despite offering valuable empirical insights into relationships among environmental experiences, artistic creation, perceived emotional healing, and perceived personal growth in outdoor landscape sketching courses in higher art education, this study has several limitations that warrant consideration.

First, the sample was drawn from three higher education institutions in China and consisted exclusively of art and design students. Although this population was appropriate for the research context, this may constrain the generalizability of the findings across broader educational and cultural contexts. Future studies should include more diverse samples across institutions, regions, and academic disciplines.

Second, the cross-sectional design limits the ability to examine how students’ experiences develop over time or to establish temporal relationships among the study variables. Longitudinal, follow-up, or quasi-experimental studies would provide stronger evidence regarding the stability and development of the observed relationships.

Third, data collection relied predominantly on self-reported instruments, which may be subject to social desirability and recall biases. Future studies are encouraged to combine self-report measures with multiple data sources, such as peer evaluations, instructor assessments, behavioral observations, or physiological indicators. Where appropriate, future studies may also incorporate physiological indicators (e.g., cortisol levels, heart rate variability) or other objective measures to complement self-report data and provide a more comprehensive understanding of emotional experiences.

Fourth, several constructs in the proposed framework demonstrated relatively strong interrelationships, particularly perceived emotional healing and perceived personal growth. Although discriminant validity criteria were satisfied, relatively strong associations between certain constructs indicate that these concepts are perhaps theoretically related. Future studies should further examine their conceptual distinctiveness using longitudinal and multi-method approaches.

Lastly, the participants represented a non-clinical population engaged in an educational activity rather than a therapeutic intervention. Accordingly, the findings should not be generalized to clinical populations or individuals with substantially different psychological characteristics. Future studies could examine whether these associations remain consistent across individuals with varying levels of psychological well-being, thereby extending understanding of the potential educational and psychological value of outdoor landscape sketching.

## Data Availability

The raw data supporting the conclusions of this article will be made available by the authors, without undue reservation.
